# Case report: (Pre)syncopal symptoms associated with a negative internal jugular venous pressure

**DOI:** 10.3389/fphys.2014.00317

**Published:** 2014-08-21

**Authors:** Niels D. Olesen, Johannes J. van Lieshout, James P. Fisher, Thomas Seifert, Henning B. Nielsen, Niels H. Secher

**Affiliations:** ^1^Department of Anesthesia, Rigshospitalet, University of CopenhagenCopenhagen, Denmark; ^2^Department of Internal Medicine, Academic Medical Centre, University of AmsterdamAmsterdam, Netherlands; ^3^Laboratory for Clinical Cardiovascular Physiology, Centre for Heart Failure Research, Academic Medical CentreAmsterdam, Netherlands; ^4^MRC/Arthritis Research UK Centre for Musculoskeletal Ageing Research, School of Life Sciences, The Medical School, University of Nottingham, Queen's Medical CentreNottingham, UK; ^5^School of Sport, Exercise & Rehabilitation Sciences, College of Life and Environmental SciencesUniversity of Birmingham, UK

**Keywords:** (pre)syncope, internal jugular vein, venous pressure, cerebrovascular circulation, exercise

## Abstract

A siphon is suggested to support cerebral blood flow but appears not to be established because internal jugular venous (IJV) pressure is close to zero in upright humans. Thus, in eleven young healthy males, IJV pressure was 9 ± 1 mmHg (mean ± *SE*) when supine and fell to 3 ± 1 mmHg when seated, and middle cerebral artery mean blood velocity (MCA V_mean_; *P* < 0.007) and the near-infrared spectroscopy-determined frontal lobe oxygenation (S_c_O_2_; *P* = 0.028) also decreased. Another subject, however, developed (pre)syncopal symptoms while seated and his IJV pressure decreased to −17 mmHg. Furthermore, his MCA V_mean_ decreased and yet within the time of observation S_c_O_2_ was not necessarily affected. These findings support the hypothesis that a negative IJV pressure that is a prerequisite for creation of a siphon provokes venous collapse inside the dura, and thereby limits rather than supports CBF.

## Introduction

Regulation of cerebral blood flow (CBF) is viewed from perspective of the arterial inflow: it is stabilized by “autoregulation” maintaining CBF despite changes in mean arterial pressure (MAP), responds to the arterial carbon dioxide tension (P_a_CO_2_), and might be modulated by sympathetic activity (Paulson et al., 1990). A venous influence on CBF is often neglected, but two views have been advanced. One contention holds that a siphon supports CBF when the head is above the level of the heart (Badeer, 1988, 1997; Hicks and Badeer, 1989, 1992). A siphon encompasses a tube with an ascending and a descending limb, of any shape in a gravitational field and requires only that the outlet is positioned lower than the inlet. For the brain, the arterial and venous system could represent the respective ascending and descending limbs of a siphon. In this inverted U-tube arrangement the heart does not need to overcome the hydrostatic influence on blood to serve the brain but needs only to overcome vascular resistance represented largely by the small vessels within the brain, even in an animal with a long neck such as the giraffe. If a siphon is established to support CBF, venous pressure should be negative (by ~ −100 mmHg for the giraffe, depending of the length of its neck) and in humans for whom the distance from the right atrium to the cerebrum is ~25 cm, venous pressure at the base of the brain should be ~ −19 mmHg.

The other argument holds that a siphon cannot be established since neck veins collapse in an upright position; their pressure approximates tissue pressure of about zero (Dawson et al., 2004; Brondum et al., 2009). According to that view, CBF is supported by arterial pressure and the carotid baroreceptors are positioned ideally to secure blood pressure at the level of the brain. In support of that concept, MAP of the giraffe is approximately twice that of humans, making MAP at the level of the brain (bMAP) similar (Goetz et al., 1960; Van Citters et al., 1966; Hargens et al., 1987; Brondum et al., 2009; Petersen et al., 2013). Also a negative venous pressure at the level of the neck would be transmitted to subdural veins and likely make them collapse impeding a siphon mechanism (Yada et al., 1973; Johnston and Rowan, 1974). This study presents a subject who developed a marked decrease in internal jugular venous (IJV) pressure when seated which is a prerequisite if a siphon should be established but since he developed (pre)syncopal symptoms, the observation indicates that a negative IJV pressure limits rather than supports CBF.

## Materials and methods

Twelve healthy male subjects (age 22 ± 1 years (mean ± *SE*), height 185 ± 2 cm; weight 76 ± 2 kg) provided informed written and oral consent to a study where cerebral perfusion and metabolism during exercise were compared among young and old subjects (Fisher et al., 2013) as approved by the ethical committee of Copenhagen (H-1-2010-141) in accordance with the Declaration of Helsinki. Data for the subject who developed pre-syncopal symptoms when seated (20 years; height 1.90 m; weight 70 kg) were excluded from the report. No subject was taking any prescribed or over-the-counter medication and no one had a history of cardiovascular, metabolic, or neurological disease. All subjects were recreationally active and no subject had recently experienced a prolonged period of physical inactivity (e.g., bed rest). The subjects were requested to abstain from strenuous physical activity on the day before the study and to report to the laboratory (~22°C) at 8:00 am after an overnight fast.

In a slightly head-down position and after local anesthesia (lidocaine 2%), a catheter (1.6 mm; 14 gauge; ES-04706, Arrow International, PA) was inserted directly into the right IJV guided by ultrasound and its tip was advanced retrograde to the bulb of the vein where blood pressure was measured with a transducer (Edwards Life Sciences, Irving, CA) zeroed at the base of the brain. A second catheter (1.1 mm; 20 gauge) was inserted in the brachial or radial artery of the non-dominant arm for determination of P_a_CO_2_ (ABL, Radiometer, Copenhagen, Denmark) and recording of MAP by another transducer zeroed at the level of the heart. The catheter lumens were flushed (3 ml/h isotonic saline) and connected to a pressure monitoring system (Dialogue-2000, Danica, Copenhagen, Denmark).

A lead II ECG monitored heart rate (HR) and was stored along with blood pressures for offline analysis (Chart v5.2 and powerlab, AD Instruments, Bella Vista, NSW, Australia). Middle cerebral artery mean blood velocity (MCA V_mean_) was monitored by transcranial Doppler sonography through the temporal ultrasound window at a depth of 48–60 mm (Multidop X, DWL, Sipplingen, Germany). When the optimal signal-to-noise ratio was obtained, the probe (2 MHz) was secured by adhesive ultrasonic gel (Tensive, Parker Laboratories, Orange, NJ) and mounted on a headband. Changes in MCA V_mean_ reflect those in CBF (Bishop et al., 1986; Hellstrom et al., 1996), since a stable diameter of the MCA can in general be assumed (Giller et al., 1993). Frontal lobe oxygenation (S_c_O_2_) was monitored by near infrared spectroscopy (NIRS; INVOS Cerebral Oximeter, Somanetics, Troy, MI.) with an optode attached to the forehead (Madsen and Secher, 1999; Rasmussen et al., 2007). In the (pre)syncopal subject ultrasound imaging of the IJV was obtained (Site~Rite 5 Vascular access, Bard Access Systems, Salt Lake City, UT).

After instrumentation, the subjects rested for 1 h to offset arousal and nociceptive stimuli. The subjects then performed incremental ergometer cycling exercise (Fisher et al., 2013). The (pre)syncopal subject experienced (pre)syncopal symptoms whenever seated still on the ergometer. Even so, he preferred to continue the exercise, and for that purpose the protocol was adapted to progressive uninterrupted stepwise increments in workload.

We did not measure intracranial pressure (ICP) and can only estimate what ICP might have been from the IJV pressure and the hydrostatic gradient to “the middle of the brain” or approximately −10 mmHg (Chapman et al., 1990). For the supine position the ICP was estimated to be similar to the IJV pressure. Thus, an estimate of cerebral perfusion pressure (CPP) was bMAP minus the considered ICP. bMAP was taken as MAP at heart level minus the hydrostatic pressure difference calculated as the product of the gravitational acceleration *g*, length of the neck *h* and the density of blood ρ, not taking the viscous resistance and kinetic energy into account.

$$\begin{array}{l} \text{CPP}=\text{bMAP}-\text{ICP}\approx \text{MAP }-\rho \text{ g h }-\text{IJV pressure}\\ \;+\;10\text{mmHg} \end{array}$$

At supine rest we considered brain and heart to be at a similar level, but an approximately 25 cm vertical distance between the heart and the brain was measured when the subjects were seated on the ergometer. Thus, the hydrostatic pressure difference was approximately 19 mmHg.

$$\begin{array}{l} \rho \;h\;g=1.05*10^{3}\frac{kg}{m^{3}}*0.25m*9.807\frac{m}{s^{2}}\\ \;=2574\frac{kg}{ms^{2}}=19\text{mmHg} \end{array}$$

### Data analysis

In order to contrast the observations from the subject who developed (pre)syncopal symptoms to the “normal” cardiovascular responses to the upright position, values for the control subjects are presented (mean ± *SE*). The statistical analysis was performed using Sigma Plot 9.0. Differences between the supine and seated position and between seated rest and exercise at 30 W in the control subjects were calculated using paired *t*-tests, and *P* < 0.05 was considered to indicate a statistical significant difference.

## Results

### Control subjects

**Figure 1:**
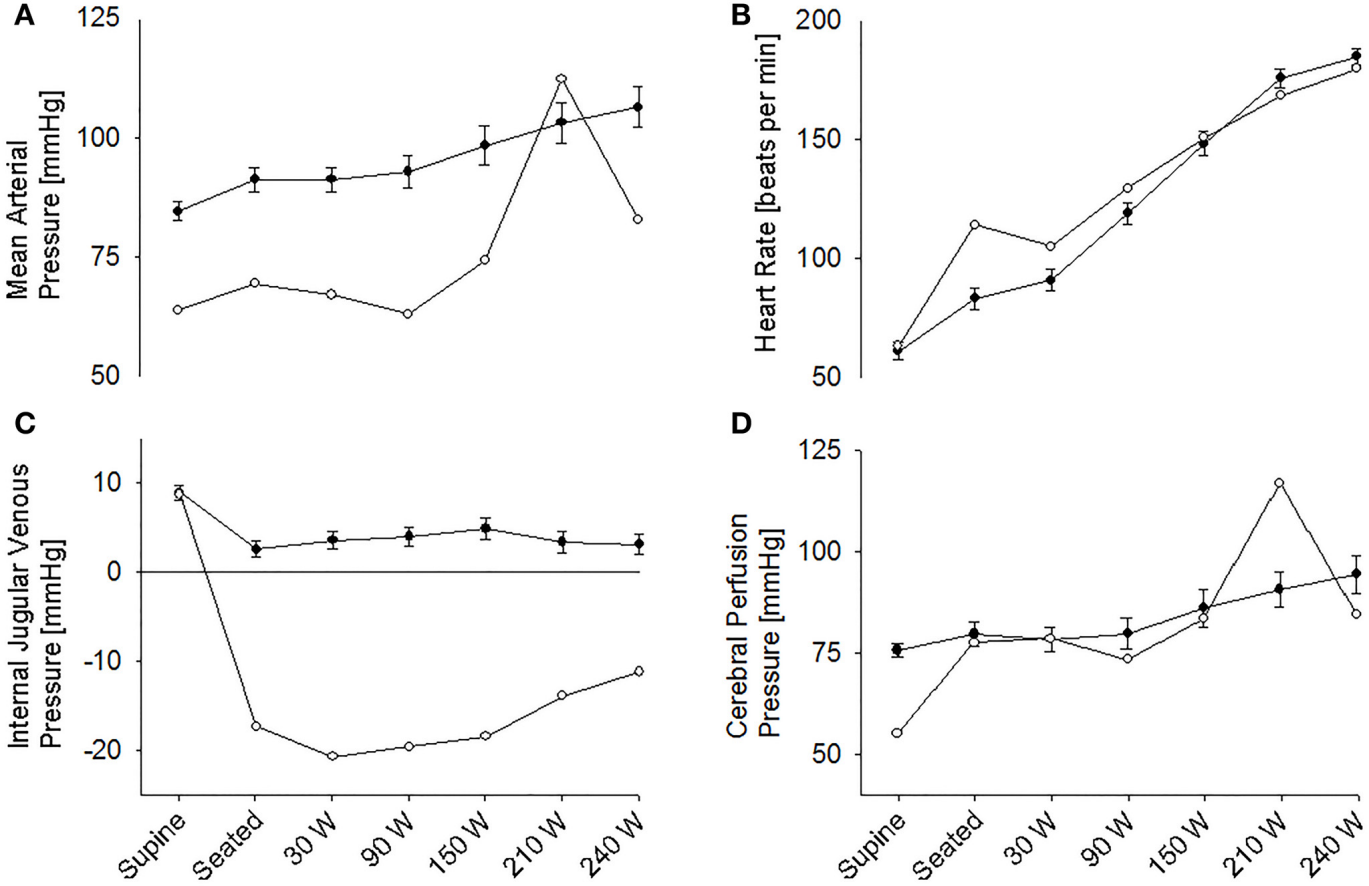
**(A)** Mean arterial pressure. **(B)** Heart rate. **(C)** Internal jugular venous pressure. **(D)** Estimated cerebral perfusion pressure during supine rest, rest while seated on the cycle ergometer, and during progressive exercise. • Control subjects (mean ± *SE* for 11 subjects). ◦ (pre)syncopal subject.

**Figure 2:**
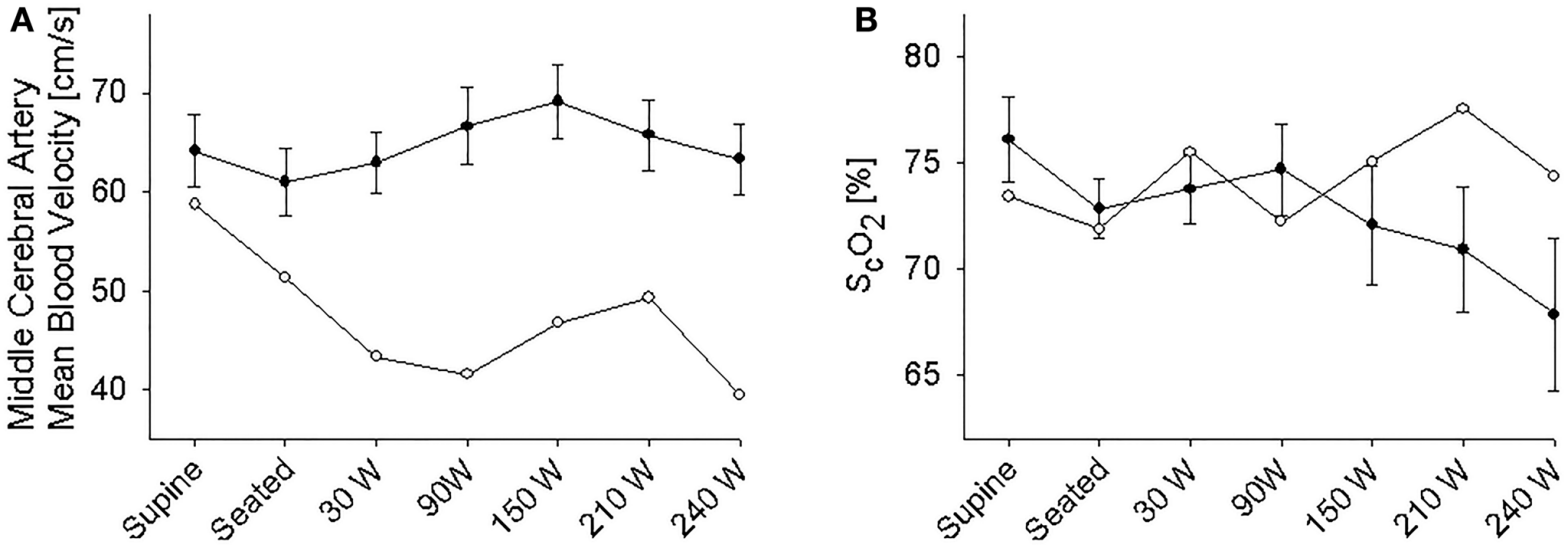
**(A)** Middle cerebral artery mean blood velocity. **(B)** Near infrared spectroscopy determined frontal lobe oxygenation (S_c_O_2_) during supine rest, rest while seated on the cycle ergometer, and during progressive exercise. • Control subjects (mean ± *SE* for 11 subjects). ◦ (pre)syncopal subject.

**Figure 3:**
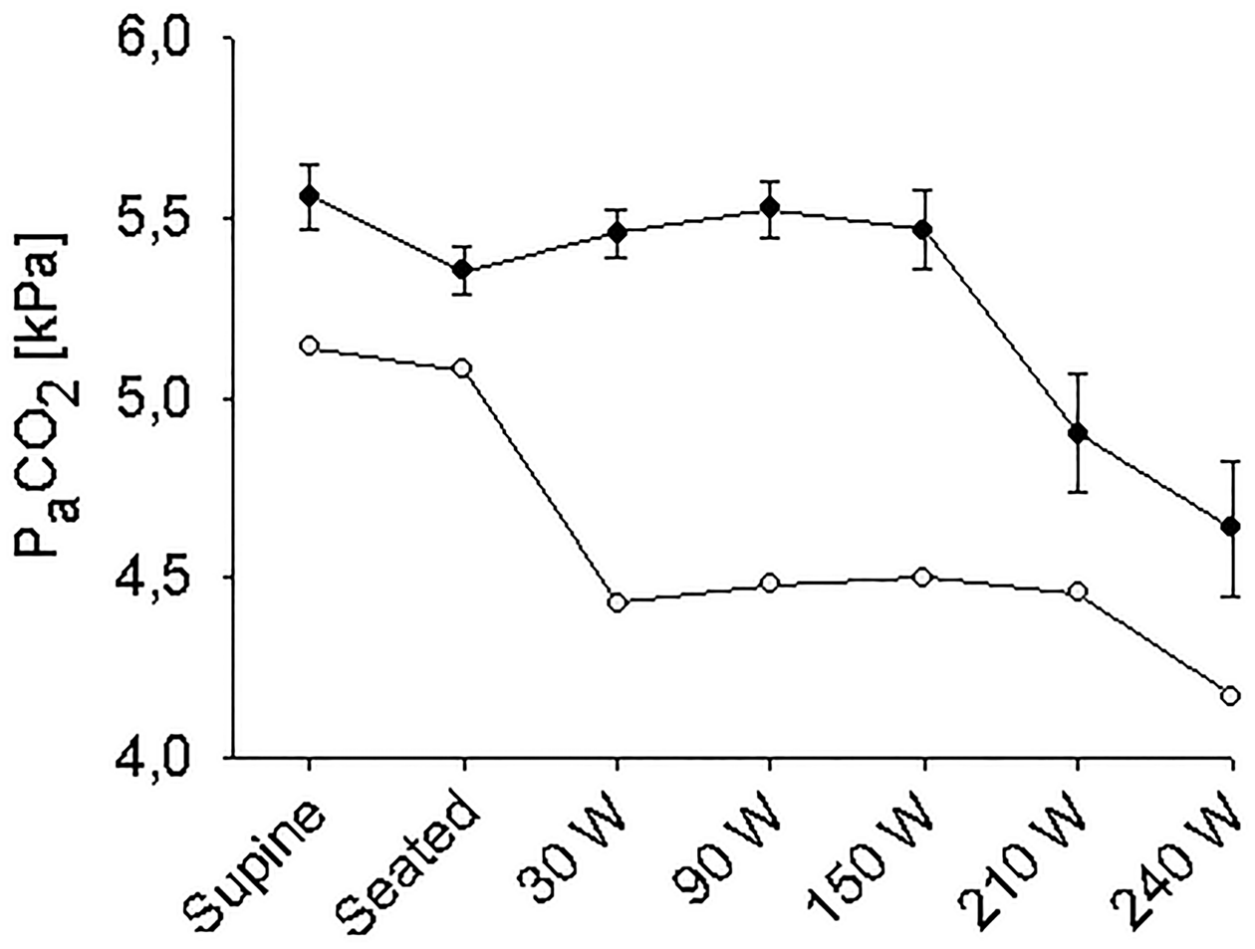
**Arterial carbon oxide tension (P_a_CO_2_) during supine rest, rest while seated on the cycle ergometer, and during progressive exercise**. • Control subjects (mean ± *SE* for 11 subjects); ◦ (pre)syncopal subject.

The supine MAP was 85 ± 2 mmHg, increasing to 91 ± 3 mmHg when the subjects were seated and likewise HR increased from 61 ± 4 to 83 ± 4 beats/min and both variables increased further with exercise intensity (Figure 1). IJV pressure decreased from 9 ± 1 to 3 ± 1 mmHg and remained stable during exercise; negative values (−1 to −2 mmHg) were measured in 5 subjects. Accordingly, estimated CPP increased from 76 ± 2 to 80 ± 3 mmHg upon siting up, followed by a progressive increase with exercise intensity as MAP became elevated. Meanwhile, S_c_O_2_ decreased from 76 ± 2 to 73 ± 1% (*P* = 0.028) and MCA V_mean_ from 64 ± 4 to 61 ± 3 cm/s (*P* < 0.007) when sitting on the ergometer; both, however, increased during low-to-moderate intensity exercise to decrease at the higher work intensities (Figure 2). Also, P_a_CO_2_ decreased when seated (from 5. 6 ± 0.1 to 5.4 ± 0.1 kPa), but increased again during light exercise, to diminish at the highest exercise intensities (Figure 3).

### The (pre)syncopal subject

**Figure 4:**
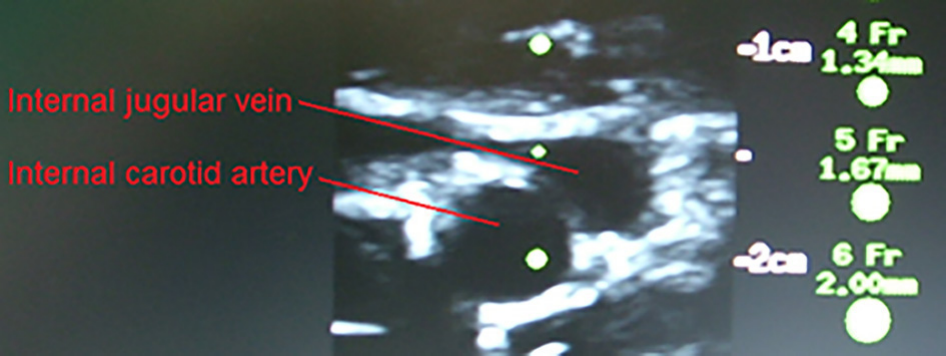
**Carotid arterial and internal jugular vein cross section for the (pre)syncopal subject when seated on the cycle ergometer**. The catheter is visualized in the internal jugular vein.

In the supine position MAP was 64 mmHg, increasing to 70 mmHg when seated and increased further during exercise. During both seated rest and 30 W exercise HR was high (Figure 1). There was only minor change in S_*c*_O_2_ (73–72%) when rising to the seated position. However, MCA V_mean_, decreased when seated (from 59 to 51 cm/s) and decreased further (to 43 cm/s) upon the start of exercise (Figure 2). Yet, MCA V_mean_ recovered to decrease again at the highest workload. In this subject P_a_CO_2_ decreased little when he was seated, while there was a marked decrease (by 13%) at the onset of exercise. P_a_CO_2_ only recuperated slightly during submaximal exercise and diminished during maximal exercise (Figure 3). Also IJV pressure decreased from 9 mmHg when supine to −17 mmHg when seated while it remained between −14 and −21 mmHg during exercise. Because of his low MAP, estimated CPP was 55 mmHg when supine, but increased when seated from 55 to 78 mmHg. Furthermore, visualization using B-mode ultrasound revealed that his right IJV maintained a cross-section area of about 19 mm^2^ at the middle of the neck when he was seated (Figure 4). (Pre)syncopal episodes manifested with concomitant reductions in MAP, HR, MCA V_mean_ with little change in S_c_O_2_ (Table 1).

**Table 1:** **Values recorded during seated rest on the cycle ergometer and during two (pre)syncopal episodes in the (pre)syncopal subject**.

	**IJV Pressure [mmHg]**	**MAP [mmHg]**	**HR [beats per min]**	**S_c_O_2_ [%]**	**MCA V_mean_ [cm/s]**
Seated rest	−17	73	112	68	51
Syncope I	−21	55 (−24%)	106 (−6%)	63 (−8%)	44 (−13%)
Syncope II	–	52 (−28%)	87 (−22%)	70 (+2%)	41 (−19%)

*Percentage changes from seated rest presented in brackets*.

## Discussion

Although abstaining from using the term siphon because the circulation is not an open system, one school considers gravitational influence on the cerebral circulation to be balanced wherefore the heart does not need to expend energy to lift blood to the level of the brain (Badeer, 1988, 1997; Hicks and Badeer, 1989, 1992). A negative venous pressure at the level of the brain is a prerequisite if CBF should be supported by a siphon when the head is lifted. On the other hand, it is also argued that a siphon cannot support CBF because, as demonstrated for both humans and in the giraffe, jugular pressure is close to zero at the level of the brain in the upright position (Dawson et al., 2004; Brondum et al., 2009). Zero pressure in the jugular vein means that the vein is collapsed and its pressure approximates that of the surrounding tissue. Thus, a significant resistance to flow develops, whereby the potential energy of blood is used to overcome friction within the vessel.

The IJV leaves the skull at the jugular foramen and therefore starts as a round structure and the level where it collapses may vary from time to time, likely depending on the position of the head. For humans a minor negative pressure (e.g., −3 mmHg) is a common observation when upright (Dawson et al., 2004) as seen for some of the control subjects, while a negative venous pressure of −21 mmHg has been reported in the neck of a giraffe (Brondum et al., 2009). According to these measurements, small pockets of blood may develop within the jugular vein that would have a “functional” length of up to 4 cm for humans and 29 cm for the giraffe and the IJV may at places maintain a cross-sectional area of 17 ± 8 mm^2^ (mean ± *SD*) in upright humans (Valdueza et al., 2000). The important point is, however, that the length of such pockets of blood is much smaller than the length of the jugular vein and with collapsed jugular veins in the upright position, the primary outflow pathway becomes the vertebral venous plexus (Epstein et al., 1970; Valdueza et al., 2000).

Yet, we present data from a subject who presented a −17 mmHg pressure in the IJV when seated and that is approximately what would be expected if the IJV remains open throughout its length and by ultrasound imaging it was demonstrated that his IJV maintained a cross section of 19 mm^2^. In contrast, in the control subjects IJV pressure remained close to zero, affirming that in general the IJV collapses in the upright position and its pressure approximates the tissue pressure. When the (pre)syncopal subject was seated, estimated CPP increased markedly compared to the control subjects, indicating that venous pressure should not be taken into consideration when estimating CPP as he developed (pre)syncopal symptoms. Thus, his (pre)syncopal symptoms were more clearly related to a low blood pressure (MAP of 64 vs. 85 ± 2 mmHg in the control subjects) than to the estimated CPP.

In support, his MCA V_mean_ decreased (59 to 51 cm/s) more markedly when moving from a supine to a seated position than in the control subjects (64 ± 4 to 61 ± 3 cm/s) indicating that establishment of a siphon was unlikely, possibly related to subdural venous collapse or increased sympathetic activity as indicated by the increase in HR (from 63 to 114 beats per min) (Van Lieshout and Secher, 2008). However, S_c_O_2_ did not drop, maybe as a result of redistribution of venous vs. arterial blood within the brain or reduction in local brain oxygen consumption. Furthermore, the decrease in MCA V_mean_when seated is unlikely to be explained by the acute change in P_a_CO_2_ as it was maintained in the (pre)syncopal subject. (Pre)syncopal episodes (Table 1) manifested with concomitant reductions in MAP, HR and MCA V_mean_ resembling the changes seen during a vasovagal syncope or a Bezold-Jarish-like reflex (Sander-Jensen et al., 1986; Van Lieshout et al., 1991; Secher et al., 1992). This subject had previously experienced (pre)syncopal symptoms during experimentation but not in everyday life, whereby the (pre)syncopal symptoms seem related to experimental settings and catheterization. IJV catheterization has been used extensively in our laboratory and we have not previously measured similar negative values and it therefore seems unlikely that the catheter should have prevented collapse of the vessel. During experimentation in the upright position development of (pre)syncopal symptoms is a calculated risk and we suggest that IJV pressure should be noted whenever symptoms appear in studies including an IJV line. Such observations would validate to what extent the present observations can be generalized.

## Conclusion

This study confirms that CBF is not influenced by siphon-like mechanism since in a seated position IJV pressure remains close to zero. However, one subject developed a marked decrease in IJV pressure when sitting up and tended to faint with more marked decrease in cerebral perfusion than for the control group, suggesting that a potentially beneficial effect on CBF by a siphon mechanism is interrupted by subdural venous collapse.

## Author contributions

Niels D. Olesen contributed to data acquisition, data analysis and writing the manuscript. Johannes J. van Lieshout, James P. Fisher and Niels H. Secher contributed to the experimental design, data acquisition and manuscript revision. Thomas Seifert and Henning B. Nielsen contributed to data acquisition and manuscript revision.

### Conflict of interest statement

The authors declare that the research was conducted in the absence of any commercial or financial relationships that could be construed as a potential conflict of interest.
